# Safe Medication Prescribing in Halton Memory Services

**DOI:** 10.1192/bjo.2024.395

**Published:** 2024-08-01

**Authors:** Divya Jain, Archisha Marya

**Affiliations:** 1 Merseycare NHS Trust, Halton, United Kingdom; 2 Liverpool University, Liverpool, United Kingdom

## Abstract

**Aims:**

- To improve safe medication prescribing by achieving a 25% improvement in the number of cases reported in the practice within six months.
- To reduce human factors contributing to medication errors to improve patient safety and quality of care.

**Methods:**

- Retrospective data collection was done for Halton and Widnes patients from March 2022 to April 2023;
- Retrospective data collection for Re-audit was done for a period between June 2023 to January 2024 to complete the audit cycle;
- Liaised with medicine management team for local practices/policies;
- Reviewed and verified Trust standardised local policies on medicine management;
- Reviewed incident data and checked processes in other teams;
- The findings were presented at the Medicine Management meeting in May 2023;
- Training on safe prescribing was delivered to the Memory team in June 2023.

**Results:**

- During the first data collection period, 14 incident forms were reported.
- During the second data collection period, 1 incident form was reported which was an administrative error.
- Prescribing errors for the first cycle accounted for 28.6%, administrative errors for 35.7%, dispensing errors for 21.4%, and other errors for 14.3%.
- Specific error types included prescribing the wrong dose/medication, medication not prescribed, medication unavailable and double prescribing.
- No incidents of restraint, seclusion, rapid tranquillisation, ambulance calls, or RIDDOR were reported.

**Conclusion:**

- Administrative errors accounted for the majority of the total reported incidents (35.7%).
- Recommendations include safe clinical practice of prescribing medication (MDT lead to update medication card and inform GP promptly).
- Other recommendations were medication card updates, aligning clinical systems, avoiding email requests and introducing Community EPMA (Trust objective to introduce EPMA to community teams in 2024/25) and to standardise procedures.
- An improvement of 92.9% in the incident reporting was found in the re-audit following a training session to the team with improved practice of no email requests or chains.
- The audit identified communication difficulties within memory services, primary care and care home.
- It also highlighted challenges related to new staff, post-MDT meetings medication card updates, prescriber preferences, geographical disparities, and doctors’ availability.

